# Unbound bilirubin: a call to reframe neonatal care and clinical decision-making

**DOI:** 10.1038/s41390-025-04667-w

**Published:** 2025-12-02

**Authors:** Thomas Hegyi

**Affiliations:** Robert Wood Johnson Medical School, Rutgers, The State University of New Jersey, New Brunswick, NJ USA

For decades, total serum bilirubin (TSB) has stood as the central biomarker for diagnosing and managing neonatal hyperbilirubinemia. Its enduring use has stemmed from historical associations with kernicterus and the relative ease of measurement. However, TSB’s role as a surrogate for bilirubin-induced neurologic dysfunction (BIND) is increasingly being questioned. A growing body of biochemical, physiological, and clinical research now shows that unbound bilirubin (Bf)—the fraction of bilirubin not bound to albumin—is the actual neurotoxic agent.^1^

It is Bf, not TSB, that more accurately reflects the risk of acute bilirubin encephalopathy and long-term neurodevelopmental impairment. With the technology to measure Bf now available and the clinical implications better understood, the time has come to reorient neonatal care to focus on Bf. Key therapeutic decisions, including medication use and total parenteral nutrition (TPN), must be informed by Bf concentrations if the goal is to minimize preventable neurologic injury.

The limited usefulness of TSB in identifying molecular and cellular injury is expected, given the mechanism behind neurotoxicity. Bilirubin is a hydrophobic, tetrapyrrolic compound formed during heme catabolism. In plasma, bilirubin is primarily carried by albumin, which binds to it with high affinity. This albumin-bilirubin complex allows safe transport to the liver for conjugation and eventual excretion. Yet, the binding between albumin and bilirubin is a dynamic equilibrium, subject to perturbation by both endogenous and exogenous influences. The small fraction of bilirubin not bound to albumin is lipid-soluble and capable of crossing the immature blood-brain barrier, where it exerts a cascade of neurotoxic effects. The strength of albumin-bilirubin binding depends on various physiological variables, including blood pH, serum albumin concentration, oxidative stress, and the presence of competitive binding molecules. Conditions commonly encountered in the neonatal intensive care unit (NICU), including prematurity, acidosis, sepsis, and hypoxia, are all known to reduce albumin binding affinity, increasing Bf even when TSB remains stable.

Exogenous compounds contribute further to this vulnerability. Free fatty acids, which rise in response to stress and nutritional interventions such as lipid-rich TPN, compete with bilirubin for albumin binding sites. Soybean oil-based lipid emulsions such as Intralipid elevate circulating free fatty acids, significantly increasing Bf levels.^2^ Conversely, fish oil-based alternatives, such as Omegaven, appear to exert less competitive displacement, presenting safer options for infants at risk. Similarly, specific pharmacological agents, such as nonsteroidal anti-inflammatory drugs and certain antibiotics, have been shown to displace bilirubin from albumin, thereby increasing Bf even in the presence of modest TSB levels. Ceftriaxone and sulfisoxazole have already been removed from the neonatal pharmacopeia for this reason, yet others, such as ibuprofen and indomethacin, remain in everyday use.

The consequences of elevated Bf are well established at the cellular level.^3^ Unbound bilirubin interferes with mitochondrial function, disrupts calcium homeostasis, promotes oxidative stress, and induces apoptosis in vulnerable neuronal populations. These effects inhibit dendritic arborization, impair neurotransmission, and compromise synaptic development. Research in both animal models and human infants has confirmed these mechanisms, which disproportionately affect preterm and sick neonates. Unlike TSB, which may remain constant despite fluctuations in binding dynamics, Bf provides a direct and timely reflection of bilirubin’s cellular bioavailability.

Clinical studies have repeatedly shown that Bf concentrations correlate more reliably with markers of neurologic dysfunction than TSB. Auditory brainstem response abnormalities, magnetic resonance imaging findings, and long-term developmental delays have all shown stronger associations with elevated Bf. Ahlfors and colleagues have confirmed that there is strong evidence that Bf measurements would improve the clinical management of newborn infants with jaundice by enhancing the identification of infants requiring treatment and reducing unnecessary interventions.^4^ Neonates with moderate TSB levels but impaired binding due to comorbidities often present with significant Bf elevations and may develop acute bilirubin encephalopathy despite TSB levels falling below conventional treatment thresholds. On the other hand, infants with high TSB but robust albumin binding often show no neurologic injury. This discrepancy highlights the need for a shift in perspective on how risk is assessed and managed.

Technological advances have now made the routine measurement of Bf possible in clinical laboratories. The peroxidase method, along with more recent fluorescent displacement techniques,^5^ can reliably quantify Bf with sufficient sensitivity and specificity for use in neonatal care. These methods meet the standards of reproducibility and precision necessary for incorporation into routine clinical workflows. Authors such as Stevenson have persuasively argued for the incorporation of Bf into routine practice, emphasizing its dynamic responsiveness to clinical status and medication effects, as well as its superior performance as a neurotoxicity marker compared to TSB.^6^

Despite the availability of technology and the clarity of scientific data, the widespread adoption of Bf monitoring remains limited. The barriers are not insurmountable but are primarily logistical and cultural. Incorporating Bf into clinical protocols will require both institutional investment and a commitment to reevaluation of long-standing practices. Point-of-care Bf testing should become a standard part of hyperbilirubinemia risk assessment, particularly in high-risk neonates. Clinical algorithms must integrate Bf thresholds in deciding the need for phototherapy or exchange transfusion, either in tandem with or in place of TSB. Pharmacologic stewardship programs should systematically evaluate medications for their potential to displace bilirubin and increase Bf, favoring safer alternatives when available. Similarly, nutrition protocols should prioritize fish-oil-based lipids in neonates with risk factors for binding impairment.

Laboratory services must also validate Bf assays in accordance with Clinical Laboratory Improvement Amendments (CLIA) and College of American Pathologists (CAP) standards, ensuring accurate and consistent results across institutions. Education is equally important. Neonatologists, nurses, pharmacists, and dietitians must receive structured training in interpreting Bf levels and understanding their clinical implications. A shared language and understanding around Bf will be critical to changing practice patterns and improving outcomes.

The neonatal brain is uniquely vulnerable to injury from unbound bilirubin, particularly in the first days of life when bilirubin production is high, albumin levels are low, and the blood-brain barrier is immature. While TSB-based management strategies have significantly reduced the incidence of kernicterus, preventable cases still occur, most often in preterm or critically ill neonates. These cases represent failures to detect or respond to elevated bilirubin (Bf) in a timely manner, not inadequacies of phototherapy or exchange transfusion. By continuing to rely primarily on TSB, clinicians risk overlooking the true biologically active fraction of bilirubin and missing the window for effective intervention.

The current evidence base, spanning biochemical studies, mechanistic investigations, and clinical outcomes research, is unambiguous. Unbound bilirubin is the proximate neurotoxic agent, and Bf is the most accurate biomarker of neurologic risk in hyperbilirubinemia. The tools to measure it exist.^4^ The impact of medications and nutrition on Bf is well documented. The argument for change is no longer theoretical.

A shift toward Bf-centric care represents an evidence-based, patient-centered refinement in neonatal medicine. The neonatal community should embrace this evolution, realigning care around the best available science. It is time to act, not in reaction to outdated thresholds but in response to the biological realities of bilirubin toxicity.
